# *tert*-Butyl 2-[4-(2-{4-[(*tert*-butoxycar­bonyl)methoxy]-3-methylphenyl}-2-propyl)-2-methylphenoxy]acetate

**DOI:** 10.1107/S1600536810023433

**Published:** 2010-06-23

**Authors:** Qamar Ali, Sammer Yousuf, Muhammad Raza Shah, Seik Weng Ng

**Affiliations:** a HEJ Research Institute of Chemistry, International Center for Chemical and Biological Sciences, University of Karachi, Karachi 75270, Pakistan; b Department of Chemistry, University of Malaya, 50603 Kuala Lumpur, Malaysia

## Abstract

In the mol­ecule of the title compound, C_29_H_40_O_6_, the carbon atom belonging to the propyl chain is connected to two aromatic rings that open up the C_ar­yl_—C—C_ar­yl_ angle to 111.5 (1)°. The four-atom –O–CH_2_–C(=O)–O– linkage between the aromatic ring and the *tert*-butyl group assumes a (−)anti-periplanar conformation for one substituent and a (−)*syn*-periplanar conformation for the other substituent; the O–C–C–O torsion angles are −173.7 (2) and −10.2 (3)°.

## Related literature

For the crystal structure of a related *V*-shaped mol­ecule, see: Shah *et al.* (2010 ▶).
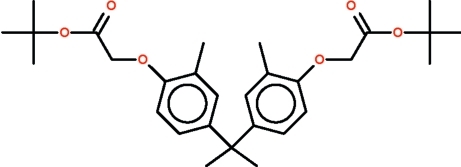


## Experimental

### 

#### Crystal data

C_29_H_40_O_6_
*M* *_r_* = 484.61
Triclinic, 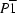

*a* = 8.3154 (6) Å
*b* = 12.5589 (8) Å
*c* = 13.9410 (9) Å
α = 101.782 (1)°
β = 97.529 (1)°
γ = 94.156 (1)°
*V* = 1405.52 (16) Å^3^
*Z* = 2
Mo *K*α radiation
μ = 0.08 mm^−1^
*T* = 293 K
0.45 × 0.35 × 0.30 mm

#### Data collection

Bruker SMART APEX diffractometer
13784 measured reflections
6428 independent reflections
4482 reflections with *I* > 2σ(*I*)
*R*_int_ = 0.021

#### Refinement

*R*[*F* ^2^ > 2σ(*F* ^2^)] = 0.060
*wR*(*F* ^2^) = 0.184
*S* = 1.03
6428 reflections
318 parameters
H-atom parameters constrained
Δρ_max_ = 0.35 e Å^−3^
Δρ_min_ = −0.19 e Å^−3^

### 

Data collection: *SMART* (Bruker, 2002 ▶); cell refinement: *SAINT* (Bruker, 2002 ▶); data reduction: *SAINT*; program(s) used to solve structure: *SHELXS97* (Sheldrick, 2008 ▶); program(s) used to refine structure: *SHELXL97* (Sheldrick, 2008 ▶); molecular graphics: *X-SEED* (Barbour, 2001 ▶); software used to prepare material for publication: *publCIF* (Westrip, 2010 ▶).

## Supplementary Material

Crystal structure: contains datablocks global, I. DOI: 10.1107/S1600536810023433/ci5103sup1.cif

Structure factors: contains datablocks I. DOI: 10.1107/S1600536810023433/ci5103Isup2.hkl

Additional supplementary materials:  crystallographic information; 3D view; checkCIF report

## Figures and Tables

**Fig. 1.**
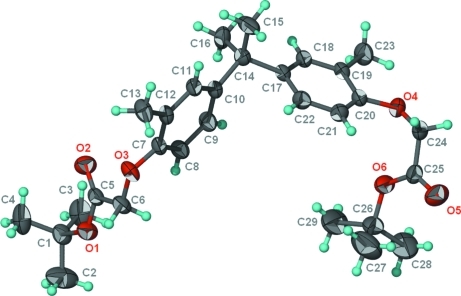
Displacement ellipsoid plot (Barbour, 2001) of C29H40O6 at the 50% probability level. H atoms are drawn as spheres of arbitrary radius.

## supplementary crystallographic information

### Comment

We are interested in the solid-state structures of *V*-shaped molecules. A recent study reported the crystal structure of 9,9-bis[4-(*tert*-butoxycarbonylmethyloxy)phenyl]fluorene, a compound used as dissolution inhibitor for protecting photosensitive poly(benzoxazole)s (Shah *et al.*, 2010). The *V* shape induced by the fluorenyl portion is now replaced by an *i*-propyl unit to furnish a similarly shaped molecule (Scheme I, Fig. 1). In the title molecule, the carbon atom belonging to the propyl chain is connected to two aromatic rings that open up the C_aryl_–*C*–C_aryl_ to 111.5 (1)°. The four-atom –O–CH_2_–C(═O)–O– chain between the aromatic ring and the *tert*-butyl group assumes a *W *shape for one substituent and a *U* shape for the other substituent [O–C–C–O torsion angle -173.7 (2)° and -10.2 (3)°].

### Experimental

2,2-Bis(4-hydroxy-3-methylphenyl)propane (0.50 g) and potassium carbonate (0.85 g) were placed in acetone (25 ml) and to this was added *tert*-butyl bromoacetate (0.75 ml, 5 mmol). The mixture was stirred at room temperature for 3 h. The solvent was evaporated under reduced pressure and the residue was dissolved in a mixture of water (50 ml) and dichloromethane (50 ml). The aqueous layer was extracted three times with dichloromethane. The combined organic phases were evaporated under reduced pressure and the solid material was recrystallized from *n*-hexane. Colourless crystals were obtained in 80% yield.

### Refinement

H atoms were placed in calculated positions [C–H = 0.93–0.97 Å] and were included in the refinement in the riding model approximation, with *U_iso_*(H) = 1.2–1.5*U_eq_*(C).

### Crystal data

**Table:**

C_29_H_40_O_6_	*Z* = 2
*M**_r_* = 484.61	*F*(000) = 524
Triclinic, *P*1	*D*_x_ = 1.145 Mg m^−^^3^
Hall symbol: -P 1	Mo *K*α radiation, λ = 0.71073 Å
*a* = 8.3154 (6) Å	Cell parameters from 4014 reflections
*b* = 12.5589 (8) Å	θ = 2.5–27.3°
*c* = 13.9410 (9) Å	µ = 0.08 mm^−^^1^
α = 101.782 (1)°	*T* = 293 K
β = 97.529 (1)°	Block, colourless
γ = 94.156 (1)°	0.45 × 0.35 × 0.30 mm
*V* = 1405.52 (16) Å^3^	

### Data collection

**Table:**

Bruker SMART APEX diffractometer	4482 reflections with *I* > 2σ(*I*)
Radiation source: fine-focus sealed tube	*R*_int_ = 0.021
graphite	θ_max_ = 27.5°, θ_min_ = 1.7°
ω scans	*h* = −10→10
13784 measured reflections	*k* = −16→14
6428 independent reflections	*l* = −18→17

### Refinement

**Table:**

Refinement on *F*^2^	Primary atom site location: structure-invariant direct methods
Least-squares matrix: full	Secondary atom site location: difference Fourier map
*R*[*F*^2^ > 2σ(*F*^2^)] = 0.060	Hydrogen site location: inferred from neighbouring sites
*wR*(*F*^2^) = 0.184	H-atom parameters constrained
*S* = 1.03	*w* = 1/[σ^2^(*F*_o_^2^) + (0.0989*P*)^2^ + 0.2549*P*] where *P* = (*F*_o_^2^ + 2*F*_c_^2^)/3
6428 reflections	(Δ/σ)_max_ = 0.001
318 parameters	Δρ_max_ = 0.35 e Å^−^^3^
0 restraints	Δρ_min_ = −0.19 e Å^−^^3^

### Fractional atomic coordinates and isotropic or equivalent isotropic displacement parameters (Å^2^)

**Table:**

	*x*	*y*	*z*	*U*_iso_*/*U*_eq_	
O1	0.54867 (16)	0.16145 (12)	0.44990 (9)	0.0584 (4)	
O2	0.28231 (18)	0.17998 (13)	0.40617 (11)	0.0644 (4)	
O3	0.22035 (16)	0.13010 (10)	0.58548 (10)	0.0537 (3)	
O4	0.13940 (17)	0.83903 (11)	1.01874 (10)	0.0608 (4)	
O5	0.4354 (2)	0.79162 (18)	1.21064 (11)	0.0891 (6)	
O6	0.43525 (16)	0.77133 (14)	1.04833 (10)	0.0665 (4)	
C1	0.6069 (3)	0.17993 (18)	0.35761 (14)	0.0571 (5)	
C2	0.7872 (3)	0.1715 (3)	0.3802 (2)	0.1093 (12)	
H2A	0.8337	0.2275	0.4369	0.164*	
H2B	0.8384	0.1808	0.3242	0.164*	
H2C	0.8044	0.1010	0.3939	0.164*	
C3	0.5730 (4)	0.29242 (19)	0.34262 (19)	0.0811 (7)	
H3A	0.6249	0.3465	0.3994	0.122*	
H3B	0.4575	0.2972	0.3344	0.122*	
H3C	0.6150	0.3051	0.2846	0.122*	
C4	0.5265 (4)	0.0919 (2)	0.27129 (18)	0.0882 (8)	
H4A	0.4108	0.0961	0.2625	0.132*	
H4B	0.5486	0.0216	0.2839	0.132*	
H4C	0.5685	0.1016	0.2124	0.132*	
C5	0.3936 (2)	0.16414 (14)	0.46314 (13)	0.0459 (4)	
C6	0.3813 (2)	0.14131 (16)	0.56480 (14)	0.0516 (4)	
H6A	0.4307	0.0747	0.5700	0.062*	
H6B	0.4434	0.2005	0.6146	0.062*	
C7	0.1432 (2)	0.22212 (13)	0.61535 (12)	0.0424 (4)	
C8	0.2100 (2)	0.32846 (15)	0.62278 (14)	0.0501 (4)	
H8	0.3146	0.3418	0.6082	0.060*	
C9	0.1196 (2)	0.41480 (14)	0.65211 (14)	0.0506 (4)	
H9	0.1661	0.4860	0.6585	0.061*	
C10	−0.0379 (2)	0.39793 (13)	0.67218 (12)	0.0429 (4)	
C11	−0.0997 (2)	0.29071 (14)	0.66524 (12)	0.0432 (4)	
H11	−0.2045	0.2775	0.6795	0.052*	
C12	−0.0121 (2)	0.20183 (13)	0.63788 (12)	0.0423 (4)	
C13	−0.0854 (3)	0.08640 (15)	0.63031 (16)	0.0593 (5)	
H13A	−0.1955	0.0875	0.6441	0.089*	
H13B	−0.0223	0.0540	0.6774	0.089*	
H13C	−0.0851	0.0443	0.5646	0.089*	
C14	−0.1407 (2)	0.49513 (14)	0.69162 (14)	0.0480 (4)	
C15	−0.3066 (2)	0.46110 (18)	0.7189 (2)	0.0755 (7)	
H15A	−0.3641	0.4036	0.6669	0.113*	
H15B	−0.3691	0.5228	0.7274	0.113*	
H15C	−0.2904	0.4356	0.7796	0.113*	
C16	−0.1702 (3)	0.53592 (18)	0.59406 (15)	0.0711 (7)	
H16A	−0.0673	0.5558	0.5749	0.107*	
H16B	−0.2309	0.5985	0.6037	0.107*	
H16C	−0.2308	0.4788	0.5429	0.107*	
C17	−0.0548 (2)	0.58614 (13)	0.77766 (12)	0.0403 (4)	
C18	−0.0610 (2)	0.69584 (13)	0.77638 (12)	0.0402 (4)	
H18	−0.1110	0.7143	0.7192	0.048*	
C19	0.0042 (2)	0.77945 (13)	0.85692 (12)	0.0420 (4)	
C20	0.0800 (2)	0.75110 (14)	0.94170 (13)	0.0445 (4)	
C21	0.0898 (2)	0.64272 (16)	0.94550 (14)	0.0522 (4)	
H21	0.1415	0.6243	1.0023	0.063*	
C22	0.0220 (2)	0.56143 (15)	0.86392 (14)	0.0494 (4)	
H22	0.0281	0.4886	0.8671	0.059*	
C23	−0.0095 (3)	0.89746 (16)	0.85277 (17)	0.0642 (6)	
H23A	0.0977	0.9358	0.8633	0.096*	
H23B	−0.0696	0.9303	0.9034	0.096*	
H23C	−0.0651	0.9015	0.7890	0.096*	
C24	0.1975 (2)	0.8205 (2)	1.11273 (14)	0.0632 (6)	
H24A	0.1263	0.7618	1.1251	0.076*	
H24B	0.1881	0.8857	1.1619	0.076*	
C25	0.3708 (2)	0.79182 (16)	1.12921 (13)	0.0527 (5)	
C26	0.6032 (2)	0.7409 (2)	1.04549 (17)	0.0702 (6)	
C27	0.6246 (5)	0.6415 (3)	1.0903 (3)	0.1357 (14)	
H27A	0.6150	0.6602	1.1595	0.203*	
H27B	0.7303	0.6178	1.0827	0.203*	
H27C	0.5419	0.5836	1.0572	0.203*	
C28	0.7205 (4)	0.8347 (3)	1.1020 (3)	0.1313 (15)	
H28A	0.7017	0.8982	1.0754	0.197*	
H28B	0.8299	0.8172	1.0967	0.197*	
H28C	0.7055	0.8493	1.1704	0.197*	
C29	0.6096 (4)	0.7084 (4)	0.9371 (2)	0.1321 (15)	
H29A	0.5931	0.7701	0.9073	0.198*	
H29B	0.5257	0.6502	0.9072	0.198*	
H29C	0.7142	0.6842	0.9270	0.198*	

### Atomic displacement parameters (Å^2^)

**Table:**

	*U*^11^	*U*^22^	*U*^33^	*U*^12^	*U*^13^	*U*^23^
O1	0.0552 (8)	0.0840 (10)	0.0451 (7)	0.0226 (7)	0.0167 (6)	0.0235 (7)
O2	0.0593 (8)	0.0816 (10)	0.0560 (8)	0.0185 (7)	0.0039 (7)	0.0224 (7)
O3	0.0603 (8)	0.0434 (7)	0.0634 (8)	0.0169 (6)	0.0273 (6)	0.0100 (6)
O4	0.0636 (8)	0.0574 (8)	0.0506 (8)	0.0147 (6)	−0.0056 (6)	−0.0076 (6)
O5	0.0735 (11)	0.1484 (18)	0.0461 (9)	0.0218 (11)	0.0038 (7)	0.0219 (9)
O6	0.0487 (8)	0.1064 (12)	0.0437 (7)	0.0157 (7)	0.0103 (6)	0.0098 (7)
C1	0.0673 (12)	0.0676 (13)	0.0446 (10)	0.0186 (10)	0.0208 (9)	0.0193 (9)
C2	0.0730 (17)	0.195 (4)	0.093 (2)	0.048 (2)	0.0443 (15)	0.075 (2)
C3	0.113 (2)	0.0603 (14)	0.0764 (16)	0.0089 (13)	0.0251 (14)	0.0213 (12)
C4	0.136 (2)	0.0705 (15)	0.0585 (14)	0.0123 (15)	0.0343 (15)	0.0028 (11)
C5	0.0526 (10)	0.0408 (9)	0.0452 (9)	0.0152 (7)	0.0102 (8)	0.0055 (7)
C6	0.0563 (11)	0.0560 (11)	0.0490 (10)	0.0241 (9)	0.0171 (8)	0.0141 (8)
C7	0.0503 (9)	0.0407 (9)	0.0375 (8)	0.0122 (7)	0.0120 (7)	0.0055 (7)
C8	0.0454 (9)	0.0462 (10)	0.0595 (11)	0.0042 (8)	0.0177 (8)	0.0074 (8)
C9	0.0538 (10)	0.0344 (9)	0.0622 (11)	0.0019 (7)	0.0144 (8)	0.0049 (8)
C10	0.0462 (9)	0.0370 (8)	0.0426 (9)	0.0070 (7)	0.0067 (7)	0.0004 (7)
C11	0.0430 (9)	0.0422 (9)	0.0429 (9)	0.0042 (7)	0.0113 (7)	0.0029 (7)
C12	0.0540 (10)	0.0355 (8)	0.0372 (8)	0.0059 (7)	0.0112 (7)	0.0041 (6)
C13	0.0721 (13)	0.0401 (10)	0.0686 (13)	0.0044 (9)	0.0302 (10)	0.0066 (9)
C14	0.0489 (10)	0.0385 (9)	0.0524 (10)	0.0110 (7)	0.0031 (8)	0.0010 (7)
C15	0.0450 (11)	0.0517 (12)	0.119 (2)	0.0112 (9)	0.0138 (11)	−0.0087 (12)
C16	0.0976 (17)	0.0548 (12)	0.0504 (11)	0.0289 (11)	−0.0129 (11)	−0.0050 (9)
C17	0.0419 (8)	0.0387 (8)	0.0405 (8)	0.0093 (7)	0.0107 (7)	0.0048 (7)
C18	0.0458 (9)	0.0396 (9)	0.0369 (8)	0.0100 (7)	0.0101 (7)	0.0079 (6)
C19	0.0441 (9)	0.0388 (9)	0.0443 (9)	0.0086 (7)	0.0130 (7)	0.0061 (7)
C20	0.0413 (9)	0.0461 (9)	0.0428 (9)	0.0090 (7)	0.0075 (7)	−0.0005 (7)
C21	0.0564 (11)	0.0567 (11)	0.0432 (9)	0.0166 (8)	0.0007 (8)	0.0105 (8)
C22	0.0586 (11)	0.0391 (9)	0.0525 (10)	0.0141 (8)	0.0060 (8)	0.0129 (8)
C23	0.0833 (15)	0.0417 (10)	0.0646 (13)	0.0103 (10)	0.0068 (11)	0.0058 (9)
C24	0.0547 (11)	0.0802 (15)	0.0449 (10)	0.0143 (10)	0.0049 (8)	−0.0102 (9)
C25	0.0516 (10)	0.0627 (12)	0.0382 (9)	0.0016 (8)	0.0045 (8)	0.0010 (8)
C26	0.0450 (11)	0.0988 (18)	0.0643 (13)	0.0143 (11)	0.0131 (9)	0.0065 (12)
C27	0.140 (3)	0.116 (3)	0.163 (4)	0.069 (2)	0.040 (3)	0.029 (3)
C28	0.0605 (16)	0.132 (3)	0.174 (4)	−0.0194 (17)	0.0303 (19)	−0.027 (3)
C29	0.087 (2)	0.228 (5)	0.081 (2)	0.045 (2)	0.0380 (17)	0.007 (2)

### Geometric parameters (Å, °)

**Table:**

O1—C5	1.328 (2)	C13—H13C	0.96
O1—C1	1.488 (2)	C14—C15	1.533 (3)
O2—C5	1.194 (2)	C14—C17	1.534 (2)
O3—C7	1.379 (2)	C14—C16	1.545 (3)
O3—C6	1.409 (2)	C15—H15A	0.96
O4—C20	1.384 (2)	C15—H15B	0.96
O4—C24	1.407 (3)	C15—H15C	0.96
O5—C25	1.191 (2)	C16—H16A	0.96
O6—C25	1.298 (2)	C16—H16B	0.96
O6—C26	1.478 (2)	C16—H16C	0.96
C1—C4	1.502 (3)	C17—C18	1.386 (2)
C1—C2	1.507 (3)	C17—C22	1.389 (2)
C1—C3	1.511 (3)	C18—C19	1.391 (2)
C2—H2A	0.96	C18—H18	0.93
C2—H2B	0.96	C19—C20	1.389 (2)
C2—H2C	0.96	C19—C23	1.507 (3)
C3—H3A	0.96	C20—C21	1.381 (3)
C3—H3B	0.96	C21—C22	1.389 (3)
C3—H3C	0.96	C21—H21	0.93
C4—H4A	0.96	C22—H22	0.93
C4—H4B	0.96	C23—H23A	0.96
C4—H4C	0.96	C23—H23B	0.96
C5—C6	1.517 (3)	C23—H23C	0.96
C6—H6A	0.97	C24—C25	1.510 (3)
C6—H6B	0.97	C24—H24A	0.97
C7—C12	1.387 (2)	C24—H24B	0.97
C7—C8	1.387 (3)	C26—C28	1.488 (4)
C8—C9	1.388 (3)	C26—C29	1.492 (4)
C8—H8	0.93	C26—C27	1.517 (5)
C9—C10	1.385 (3)	C27—H27A	0.96
C9—H9	0.93	C27—H27B	0.96
C10—C11	1.386 (2)	C27—H27C	0.96
C10—C14	1.540 (2)	C28—H28A	0.96
C11—C12	1.392 (2)	C28—H28B	0.96
C11—H11	0.93	C28—H28C	0.96
C12—C13	1.509 (2)	C29—H29A	0.96
C13—H13A	0.96	C29—H29B	0.96
C13—H13B	0.96	C29—H29C	0.96
			
C5—O1—C1	122.52 (14)	C14—C15—H15B	109.5
C7—O3—C6	119.71 (14)	H15A—C15—H15B	109.5
C20—O4—C24	119.61 (16)	C14—C15—H15C	109.5
C25—O6—C26	123.23 (16)	H15A—C15—H15C	109.5
O1—C1—C4	109.56 (18)	H15B—C15—H15C	109.5
O1—C1—C2	101.98 (16)	C14—C16—H16A	109.5
C4—C1—C2	111.8 (2)	C14—C16—H16B	109.5
O1—C1—C3	109.83 (17)	H16A—C16—H16B	109.5
C4—C1—C3	111.7 (2)	C14—C16—H16C	109.5
C2—C1—C3	111.5 (2)	H16A—C16—H16C	109.5
C1—C2—H2A	109.5	H16B—C16—H16C	109.5
C1—C2—H2B	109.5	C18—C17—C22	117.09 (15)
H2A—C2—H2B	109.5	C18—C17—C14	121.98 (15)
C1—C2—H2C	109.5	C22—C17—C14	120.75 (15)
H2A—C2—H2C	109.5	C17—C18—C19	122.83 (15)
H2B—C2—H2C	109.5	C17—C18—H18	118.6
C1—C3—H3A	109.5	C19—C18—H18	118.6
C1—C3—H3B	109.5	C20—C19—C18	118.19 (15)
H3A—C3—H3B	109.5	C20—C19—C23	120.99 (16)
C1—C3—H3C	109.5	C18—C19—C23	120.81 (16)
H3A—C3—H3C	109.5	C21—C20—O4	124.86 (16)
H3B—C3—H3C	109.5	C21—C20—C19	120.66 (16)
C1—C4—H4A	109.5	O4—C20—C19	114.47 (15)
C1—C4—H4B	109.5	C20—C21—C22	119.52 (16)
H4A—C4—H4B	109.5	C20—C21—H21	120.2
C1—C4—H4C	109.5	C22—C21—H21	120.2
H4A—C4—H4C	109.5	C21—C22—C17	121.70 (16)
H4B—C4—H4C	109.5	C21—C22—H22	119.2
O2—C5—O1	127.28 (17)	C17—C22—H22	119.2
O2—C5—C6	125.41 (17)	C19—C23—H23A	109.5
O1—C5—C6	107.31 (15)	C19—C23—H23B	109.5
O3—C6—C5	113.92 (15)	H23A—C23—H23B	109.5
O3—C6—H6A	108.8	C19—C23—H23C	109.5
C5—C6—H6A	108.8	H23A—C23—H23C	109.5
O3—C6—H6B	108.8	H23B—C23—H23C	109.5
C5—C6—H6B	108.8	O4—C24—C25	116.98 (17)
H6A—C6—H6B	107.7	O4—C24—H24A	108.1
O3—C7—C12	114.91 (15)	C25—C24—H24A	108.1
O3—C7—C8	124.60 (16)	O4—C24—H24B	108.1
C12—C7—C8	120.48 (15)	C25—C24—H24B	108.1
C7—C8—C9	119.50 (17)	H24A—C24—H24B	107.3
C7—C8—H8	120.2	O5—C25—O6	126.65 (19)
C9—C8—H8	120.2	O5—C25—C24	120.14 (18)
C10—C9—C8	121.84 (16)	O6—C25—C24	113.20 (16)
C10—C9—H9	119.1	O6—C26—C28	109.1 (2)
C8—C9—H9	119.1	O6—C26—C29	102.79 (19)
C9—C10—C11	116.96 (15)	C28—C26—C29	116.1 (3)
C9—C10—C14	119.77 (15)	O6—C26—C27	109.7 (2)
C11—C10—C14	123.03 (15)	C28—C26—C27	110.2 (3)
C10—C11—C12	123.05 (16)	C29—C26—C27	108.5 (3)
C10—C11—H11	118.5	C26—C27—H27A	109.5
C12—C11—H11	118.5	C26—C27—H27B	109.5
C7—C12—C11	118.11 (15)	H27A—C27—H27B	109.5
C7—C12—C13	120.80 (15)	C26—C27—H27C	109.5
C11—C12—C13	121.08 (16)	H27A—C27—H27C	109.5
C12—C13—H13A	109.5	H27B—C27—H27C	109.5
C12—C13—H13B	109.5	C26—C28—H28A	109.5
H13A—C13—H13B	109.5	C26—C28—H28B	109.5
C12—C13—H13C	109.5	H28A—C28—H28B	109.5
H13A—C13—H13C	109.5	C26—C28—H28C	109.5
H13B—C13—H13C	109.5	H28A—C28—H28C	109.5
C15—C14—C17	106.90 (16)	H28B—C28—H28C	109.5
C15—C14—C10	111.80 (15)	C26—C29—H29A	109.5
C17—C14—C10	111.47 (14)	C26—C29—H29B	109.5
C15—C14—C16	108.36 (18)	H29A—C29—H29B	109.5
C17—C14—C16	111.53 (15)	C26—C29—H29C	109.5
C10—C14—C16	106.79 (15)	H29A—C29—H29C	109.5
C14—C15—H15A	109.5	H29B—C29—H29C	109.5
			
C5—O1—C1—C4	−62.9 (2)	C15—C14—C17—C18	−95.7 (2)
C5—O1—C1—C2	178.6 (2)	C10—C14—C17—C18	141.89 (16)
C5—O1—C1—C3	60.2 (3)	C16—C14—C17—C18	22.6 (2)
C1—O1—C5—O2	0.6 (3)	C15—C14—C17—C22	79.1 (2)
C1—O1—C5—C6	179.73 (16)	C10—C14—C17—C22	−43.3 (2)
C7—O3—C6—C5	−80.1 (2)	C16—C14—C17—C22	−162.58 (17)
O2—C5—C6—O3	5.4 (3)	C22—C17—C18—C19	−0.6 (3)
O1—C5—C6—O3	−173.69 (15)	C14—C17—C18—C19	174.44 (15)
C6—O3—C7—C12	−177.31 (15)	C17—C18—C19—C20	0.6 (3)
C6—O3—C7—C8	3.2 (3)	C17—C18—C19—C23	−178.41 (17)
O3—C7—C8—C9	178.89 (17)	C24—O4—C20—C21	−8.0 (3)
C12—C7—C8—C9	−0.6 (3)	C24—O4—C20—C19	170.98 (16)
C7—C8—C9—C10	−1.5 (3)	C18—C19—C20—C21	0.0 (3)
C8—C9—C10—C11	2.3 (3)	C23—C19—C20—C21	178.97 (18)
C8—C9—C10—C14	−172.32 (17)	C18—C19—C20—O4	−179.11 (15)
C9—C10—C11—C12	−1.1 (3)	C23—C19—C20—O4	−0.1 (2)
C14—C10—C11—C12	173.34 (16)	O4—C20—C21—C22	178.45 (17)
O3—C7—C12—C11	−177.78 (14)	C19—C20—C21—C22	−0.5 (3)
C8—C7—C12—C11	1.8 (3)	C20—C21—C22—C17	0.6 (3)
O3—C7—C12—C13	0.7 (2)	C18—C17—C22—C21	0.0 (3)
C8—C7—C12—C13	−179.79 (18)	C14—C17—C22—C21	−175.09 (17)
C10—C11—C12—C7	−0.9 (3)	C20—O4—C24—C25	82.9 (2)
C10—C11—C12—C13	−179.34 (17)	C26—O6—C25—O5	1.7 (4)
C9—C10—C14—C15	−176.28 (18)	C26—O6—C25—C24	−179.7 (2)
C11—C10—C14—C15	9.4 (3)	O4—C24—C25—O5	168.5 (2)
C9—C10—C14—C17	−56.7 (2)	O4—C24—C25—O6	−10.2 (3)
C11—C10—C14—C17	129.00 (18)	C25—O6—C26—C28	−65.1 (3)
C9—C10—C14—C16	65.4 (2)	C25—O6—C26—C29	171.0 (3)
C11—C10—C14—C16	−109.0 (2)	C25—O6—C26—C27	55.7 (3)

## References

[bb1] Barbour, L. J. (2001). *J. Supramol. Chem.* **1**, 189–191.

[bb2] Bruker (2002). *SMART* and *SAINT* Bruker AXS Inc., Madison, Wisconsin, USA.

[bb3] Shah, K., Yousuf, S., Raza Shah, M. & Ng, S. W. (2010). *Acta Cryst.* E**66**, o1705. 10.1107/S1600536810022579 PMC3006837 21587925

[bb4] Sheldrick, G. M. (2008). *Acta Cryst.* A**64**, 112–122. 10.1107/S0108767307043930 18156677

[bb5] Westrip, S. P. (2010). *publCIF* In preparation.

